# Supplementary material for: Diffusion of Water Molecules on the Surface of Silica Nanoparticles—Insights from Nuclear Magnetic Resonance Relaxometry
Source: J Phys Chem B. 2024 Jan 31;128(6):1535–43. doi: 10.1021/acs.jpcb.3c06451 (PMC10875636; doi:10.1021/acs.jpcb.3c06451)
Supplement: Supplementary file 1 — jp3c06451_si_001.pdf [file jp3c06451_si_001.pdf]

Supporting Information

Diffusion of Water Molecules on the Surface of Silica Nanoparticles – Insight from Nuclear Magnetic Resonance Relaxometry

*Aleksandra Stankiewicz, Adam Kasparek, Elzbieta Masiewicz, Danuta Kruk**

Department of Physics and Biophysics, University of Warmia & Mazury in Olsztyn,

Oczapowskiego 4, 10-719 Olsztyn, Poland

Corresponding author: danuta.kruk@uwm.edu.pl

Examples of ^{1}H magnetization curves for water dispersions of silica nanoparticles.

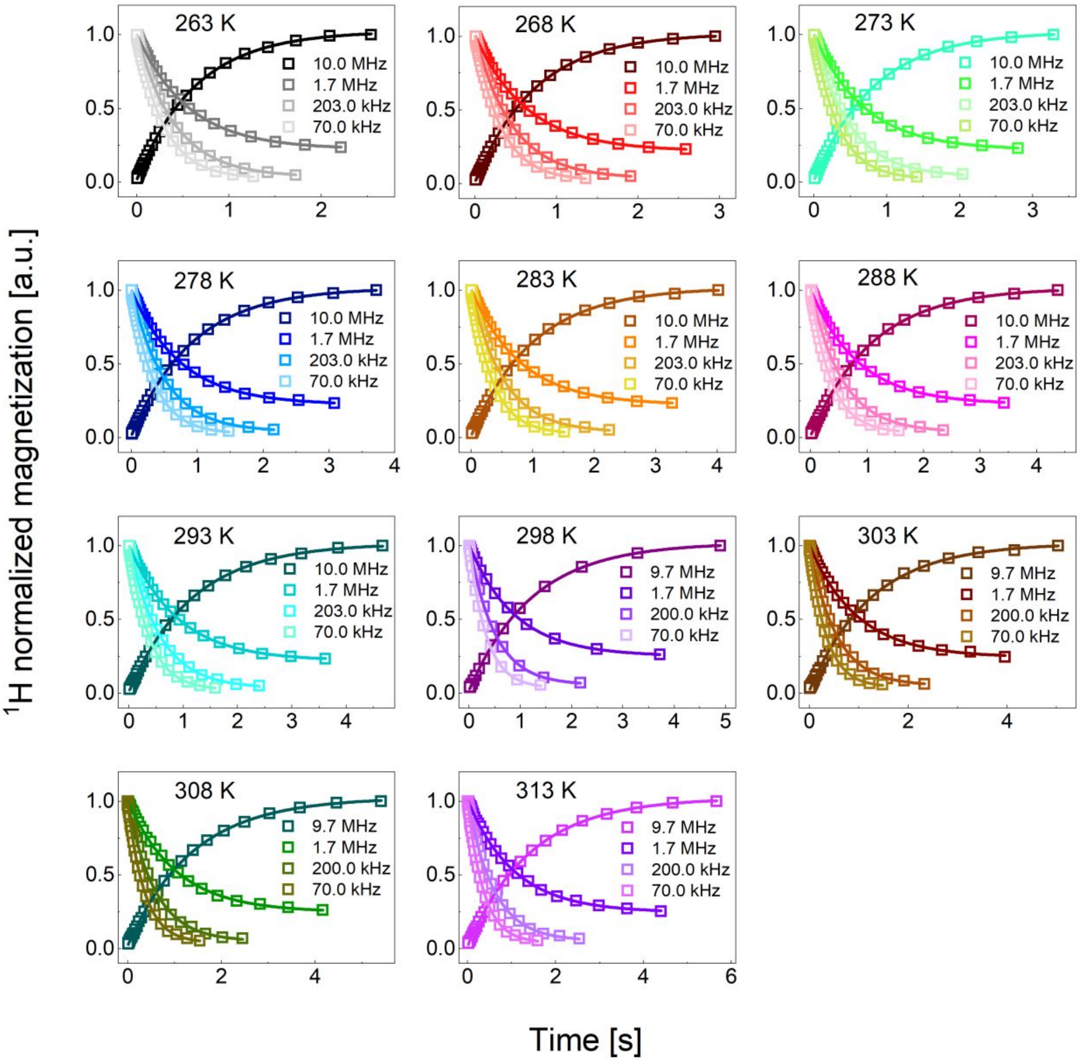

Figure S1. ^{1}H magnetization curves for water dispersion of silica nanoparticles of 45 nm diameter.

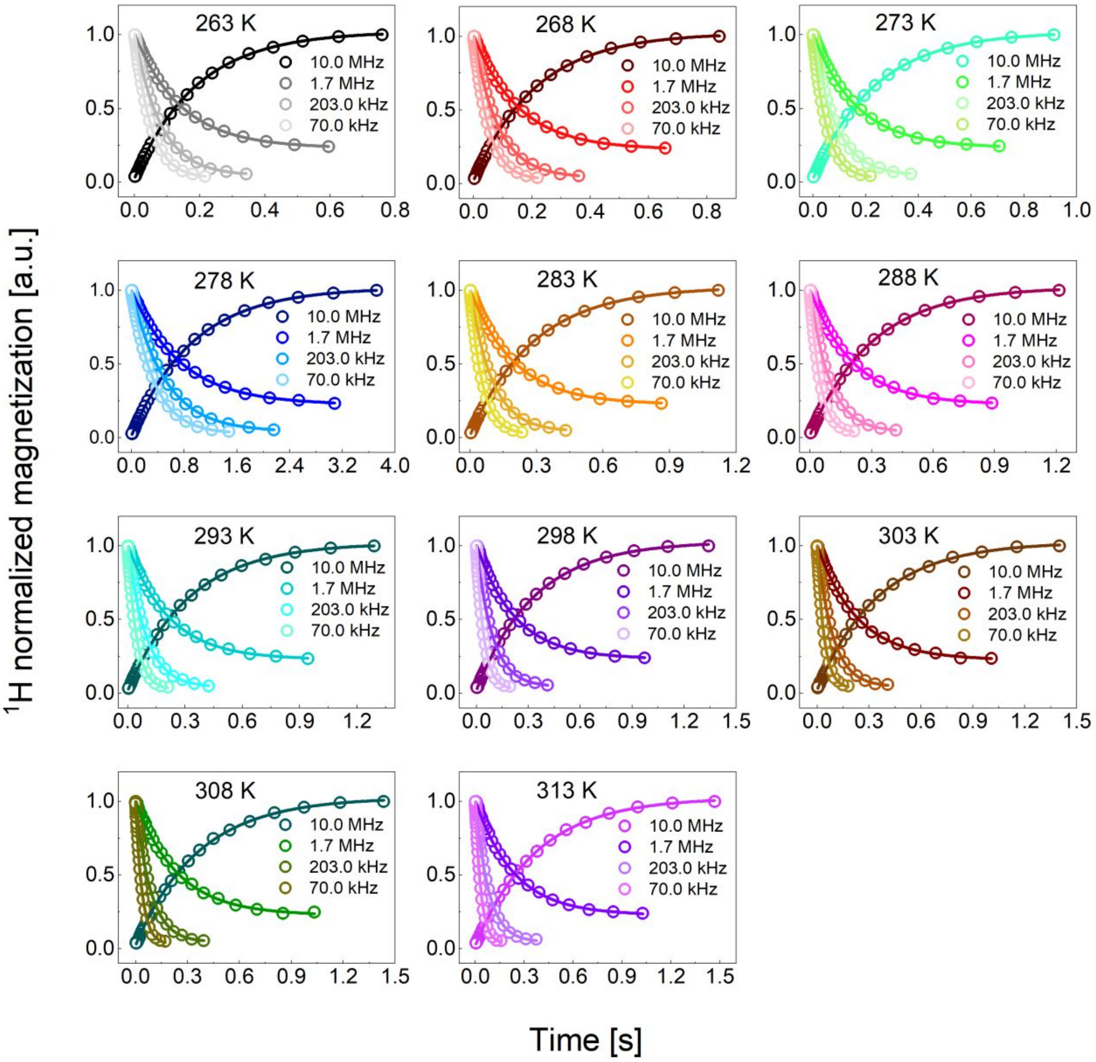

Figure S2. [1]H magnetization curves for water dispersion of silica nanoparticles of 25 nm diameter.

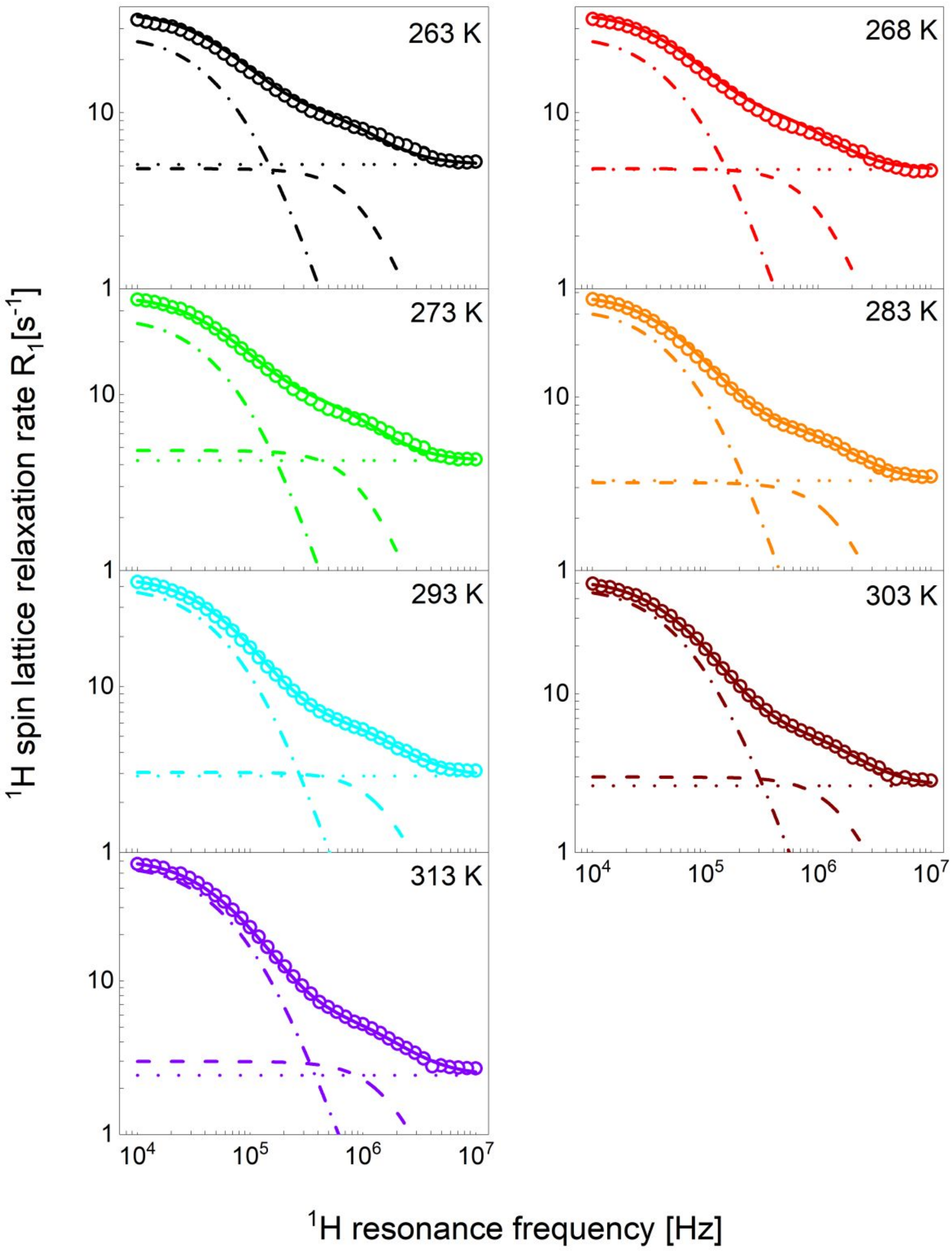

Figure S3. ^{1}H spin-lattice relaxation rates for water dispersion of silica nanoparticles of 25 nm diameter, 2.44×10^{-2} mmol/dm^3 concentration. Solid lines – fits in term of Equation 6

decomposed into a relaxation contribution associated with two-dimensional translation diffusion (dashed-dotted lines), a relaxation contribution expressed in terms of Lorentzian spectral densities (dashed lines) and a frequency independent term (dotted lines).

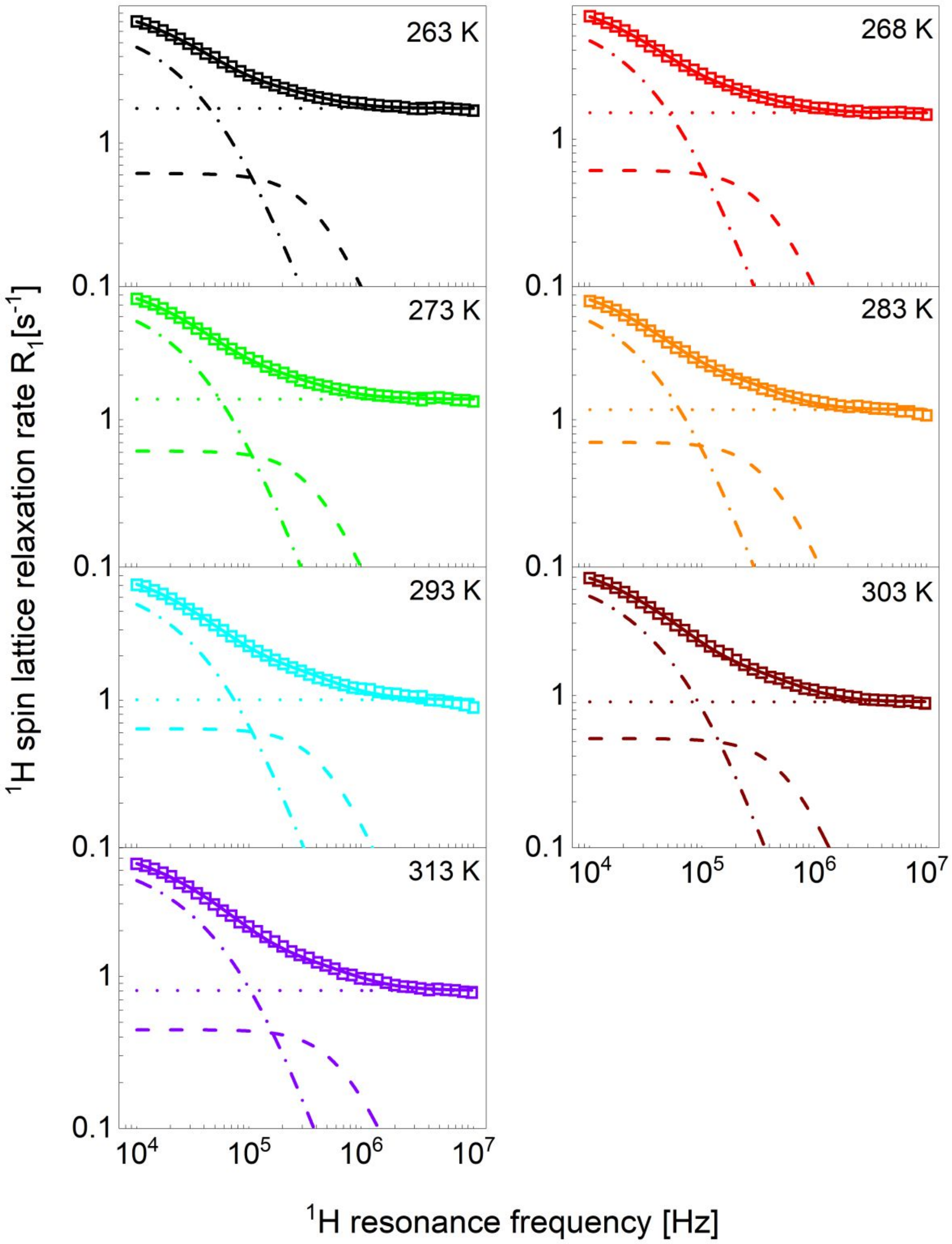

Figure S4. ^{1}H spin-lattice relaxation rates for water dispersion of silica nanoparticles of 45 nm diameter, 4.62×10^{-3} mmol/dm^3 concentration. Solid lines – fits in term of Equation 6 decomposed into a relaxation contribution associated with two-dimensional translation diffusion (dashed-dotted lines), a relaxation contribution expressed in terms of Lorentzian spectral densities (dashed lines) and a frequency independent term (dotted lines).
